# A Molecular Phylogeny for the Leaf-Roller Moths (Lepidoptera: Tortricidae) and Its Implications for Classification and Life History Evolution

**DOI:** 10.1371/journal.pone.0035574

**Published:** 2012-04-19

**Authors:** Jerome C. Regier, John W. Brown, Charles Mitter, Joaquín Baixeras, Soowon Cho, Michael P. Cummings, Andreas Zwick

**Affiliations:** 1 Department of Entomology, University of Maryland, College Park, Maryland, United States of America; 2 Institute for Bioscience and Biotechnology Research, College Park, Maryland, United States of America; 3 Systematic Entomology Laboratory, Agricultural Research Service, United States Department of Agriculture, Beltsville, Maryland, United States of America; 4 Cavanilles Institute of Biodiversity and Evolutionary Biology, University of Valencia, Valencia, Spain; 5 Department of Plant Medicine, Chungbuk National University, Cheongju, Korea; 6 Laboratory of Molecular Evolution, Center for Bioinformatics and Computational Biology, University of Maryland, College Park, Maryland, United States of America; 7 Department of Entomology, State Museum of Natural History, Stuttgart, Germany; Biodiversity Insitute of Ontario - University of Guelph, Canada

## Abstract

**Background:**

Tortricidae, one of the largest families of microlepidopterans, comprise about 10,000 described species worldwide, including important pests, biological control agents and experimental models. Understanding of tortricid phylogeny, the basis for a predictive classification, is currently provisional. We present the first detailed molecular estimate of relationships across the tribes and subfamilies of Tortricidae, assess its concordance with previous morphological evidence, and re-examine postulated evolutionary trends in host plant use and biogeography.

**Methodology/Principal Findings:**

We sequenced up to five nuclear genes (6,633 bp) in each of 52 tortricids spanning all three subfamilies and 19 of the 22 tribes, plus up to 14 additional genes, for a total of 14,826 bp, in 29 of those taxa plus all 14 outgroup taxa. Maximum likelihood analyses yield trees that, within Tortricidae, differ little among data sets and character treatments and are nearly always strongly supported at all levels of divergence. Support for several nodes was greatly increased by the additional 14 genes sequenced in just 29 of 52 tortricids, with no evidence of phylogenetic artifacts from deliberately incomplete gene sampling. There is strong support for the monophyly of Tortricinae and of Olethreutinae, and for grouping of these to the exclusion of Chlidanotinae. Relationships among tribes are robustly resolved in Tortricinae and mostly so in Olethreutinae. Feeding habit (internal versus external) is strongly conserved on the phylogeny. Within Tortricinae, a clade characterized by eggs being deposited in large clusters, in contrast to singly or in small batches, has markedly elevated incidence of polyphagous species. The five earliest-branching tortricid lineages are all species-poor tribes with mainly southern/tropical distributions, consistent with a hypothesized Gondwanan origin for the family.

**Conclusions/Significance:**

We present the first robustly supported phylogeny for Tortricidae, and a revised classification in which all of the sampled tribes are now monophyletic.

## Introduction

Tortricoidea, currently comprised of the single family Tortricidae, constitute one of the largest superfamilies of Lepidoptera, second only to Gelechioidea among the non-Obtectomera [1]. The nearly 10,000 described species [2] are distributed worldwide, with greatest species richness in the New World tropics. Tortricidae include numerous major pests of crops, forests, and ornamental plants [3]–[5], as well as biological control agents used successfully against invasive weeds [6]–[10]. Several tortricids have also become model organisms for the study of lepidopteran genetics, insect pheromones, and evolution [11]. A reliable classification and phylogeny are indispensable for the organization, communication and prediction of facts about such an economically important group of insects and for understanding how the traits important to their management and exploitation, particularly their host-plant ranges, evolve.

**Figure 1:**
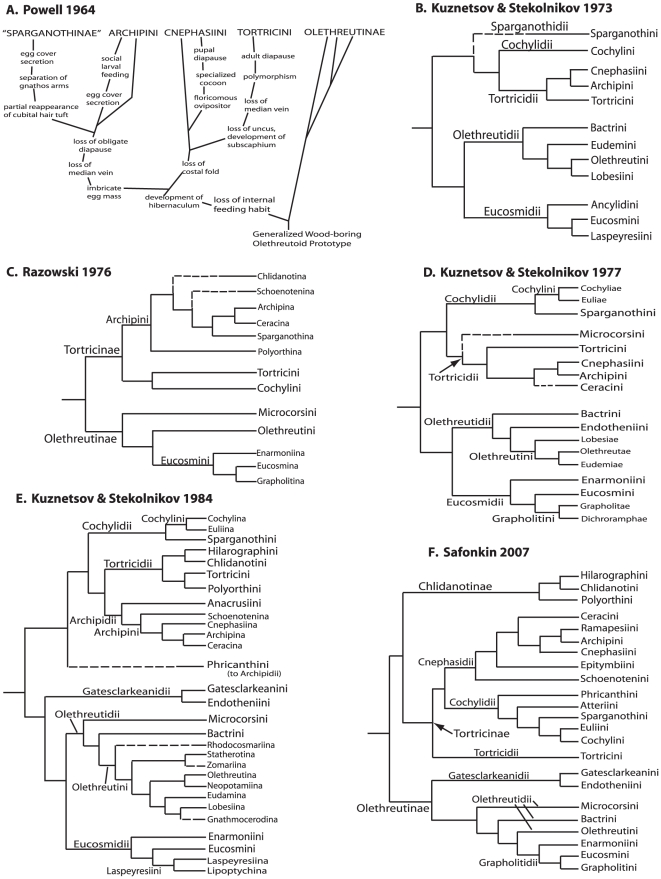
Previous hypotheses of phylogenetic relationships in Tortricidae. A. Powell (1964; [20]), B. Kuznetsov and Stekolnikov (1973; [21]), C. Razowski (1976; [23]), D. Kuznetsov and Stekolnikov (1977; [17]), E. Kuznetsov and Stekolnikov (1984; [22]), F. Safonkin (2007; [25]). Tree figures re-drawn, but nomenclature in each case follows the original.

With minor exceptions, i.e., the exclusion of Carposinidae (Walsingham 1907) and the inclusion of several small groups from Glyphipterigidae sensu lato (e.g., *Hilarographa* and relatives [12], [13]), the circumscription/definition of Tortricoidea has remained constant for over a century. The ranks of the included taxa, however, and our understanding of relationships among the many well-defined lineages have changed extensively, particularly in the last 50 years. Many groups treated as separate families by earlier authors are now recognized as tribes or subfamilies within Tortricidae (summary in Table 1), most recently Olethreutinae [14], Chlidanotinae [15], [16], and Cochylini [17]. Although hypotheses on the phylogeny of Tortricoidea were proposed historically by Meyrick [18] and Kennel [19], the first modern phylogenetic tree was presented by Powell [20], emphasizing morphological and biological characters in a treatment of the North American tribes of Tortricinae (see Fig. 1A). Based on morphology of the male and female genitalia, with an emphasis on musculature of the male genitalia, Kuznetsov and Stekolnikov [17], [21], [22] and Razowski [23] provided phylogenies for the Palearctic Tortricidae, examining a broader range of taxa than Powell, but focusing on a narrower range of characters (Figs. 1B–1E). These authors, however, do not identify the characters that support specific branches, nor do they differentiate between synapomorphic and symplesiomorphic states, and many of their taxa are defined by hypothesized shared losses. Horak and R. Brown [24], in a review of the morphology, biology, and biogeography of tortricid tribes, divided the family into the three currently recognized subfamilies, and presented a detailed working hypothesis of phylogeny for Olethreutinae ([24] their figure 1.2.5). Safonkin [25], in a review of the phylogenetic distribution of tortricid pheromones, presented the first phylogenetic tree to include three subfamilies (Fig. 1F), with relationships among tribes following Kuznetsov and Stekolnikov [22].

**Table 1:** Groups previously considered families by one or more tortricid workers.

Historical family name	Current name (Horak & Brown [24] )
Olethreutidae	Olethreutinae
Chlidanotidae	Chlidanotinae
Sparganothidae	Sparganothini
Schoenotenidae	Schoenotenini
Ceracidae	Ceracini
Phaloniidae ( = Cochylidae)	Cochylini
Atteridae	Atteriini
Eucosmiidae	Eucosmini
Grapholitidae	Grapholitini
Melanolophidae	Olethreutinae

Our current classification stems from Horak and R. Brown ([24]; see Table 2), and has undergone only a few minor changes in the past two decades, including proposed relegation of Endotheniini to Bactrini [26], and of Gatesclarkeanini to Olethreutini [27]. Although the classification has been stable, it has become increasingly clear over the past 20 years that our understanding of tortricid phylogeny remains highly provisional (review in Powell and J. Brown [28]). While many tribes include a core monophyletic group of genera, the tribal assignments of “orphan” genera renders some of the tribes paraphyletic. The monophyly of the largest subfamily, Tortricinae, has been repeatedly doubted [24], [29]. Evidence on phylogenetic relationships among tribes within subfamilies remains very scarce, and morphological analyses, thus far, have not yielded compelling clarification. For example, although Horak [27] presented a thorough cladistic analysis of the genera of Olethreutinae of Australia, she declined to propose a revised classification based on those results because they showed little similarity to traditional and intuitive relationships that could be easily supported by synapomorphies.

**Table 2:** Three recent classifications of Tortricidae and their agreement with molecular evidence. Number of exemplars in current study is given.

Powell [96]	Horak & Brown [24]	Horak [27]	Evidence from current study
**CHLIDANOTINAE**	**CHLIDANOTINAE**	**CHLIDANOTINAE**	**CHLIDANOTINAE** (5, paraphyletic)
[not included]	Polyorthini	Polyorthini	Polyorthini (2, monophyletic)
[not included]	Chlidanotini	Chlidanotini	Chlidanotini (2, monophyletic)
Hilarographini	Hilarographini	Hilarographini	Hilarographini (1)
**TORTRICINAE**	**TORTRICINAE**	**TORTRICINAE**	**TORTRICINAE** (23, monophyletic)
[not included]	Phricanthini	Phricanthini	Phricanthini (1)
Tortricini	Tortricini	Tortricini	Tortricini (1)
Cochylidae	Cochylini	Cochylini	Cochylini (3, monophyletic).
Cnephasiini (in part)	Cnephasiini	Cnephasiini	Cnephasiini (2, monophyletic)
Cnephasiini (in part)	Euliini	Euliini	Euliini (4, paraphyletic). Here synonymized with Cochylini
[not included]	Schoenotenini	Schoenotenini	[not sampled]
[not included]	Atteriini	Atteriini	Atteriini (1)
Sparganothini/Niasomini	Sparganothini	Sparganothini	Sparganothini (3, monophyletic)
[not included]	Epitymbiini	Epitymbiini	[not sampled]
Archipini	Archipini	Archipini	Archipini (6, monophyletic)
[not included]	Ceracini	Ceracini	Ceracini (1)
**OLETHREUTINAE**	**OLETHREUTINAE**	**OLETHREUTINAE**	**OLETHREUTINAE** (24, monophyletic)
[not included]	Microcorsini	Microcorsini	Microcorsini (1)
Olethreutini	Bactrini	Bactrini	Bactrini (1). Here synonymized with Olethreutini
Olethreutini	Endothenini	Bactrini	Endothenini (1). Here synonymized with Olethreutini
[not included]	Gatesclarkeanini	Olethreutini	[not sampled]
Olethreutini	Olethreutini	Olethreutini	Olethreutini (6, paraphyletic). Here broadened to include Bactrini and Endotheniini
Eucosmini	Enarmoniini	Enarmoniini	Enarmoniini (1)
Grapholitini	Grapholitini	Grapholitini	Grapholitini (6, monophyletic)
Eucosmini	Eucosmini	Eucosmini	Eucosmini (6, monophyletic)

Very recently, molecular data for small samples of Tortricidae, gathered as part of broad phylogenetic surveys across the Lepidoptera, have shown much promise for resolving relationships within tortricids [30]–[32]. The purpose of this paper, building on those preliminary findings, is to present the first detailed molecular estimate of relationships across the tribes and subfamilies of Tortricidae. Using 19 genes previously sequenced by Cho et al. [32], we expand those authors' taxon sampling from nine tortricids to 52, spanning all three subfamilies and 19 of the 22 tribes recognized by Horak and R. Brown [24]. We then review the agreement and disagreement of the molecular phylogeny with traditional morphological data and the tribal and subfamily concepts based on them. Finally, we use the new phylogeny to reconsider previous hypotheses about the evolution of host plant use in Tortricidae [33].

## Materials and Methods

### Taxon and gene sampling

**Figure 2:**
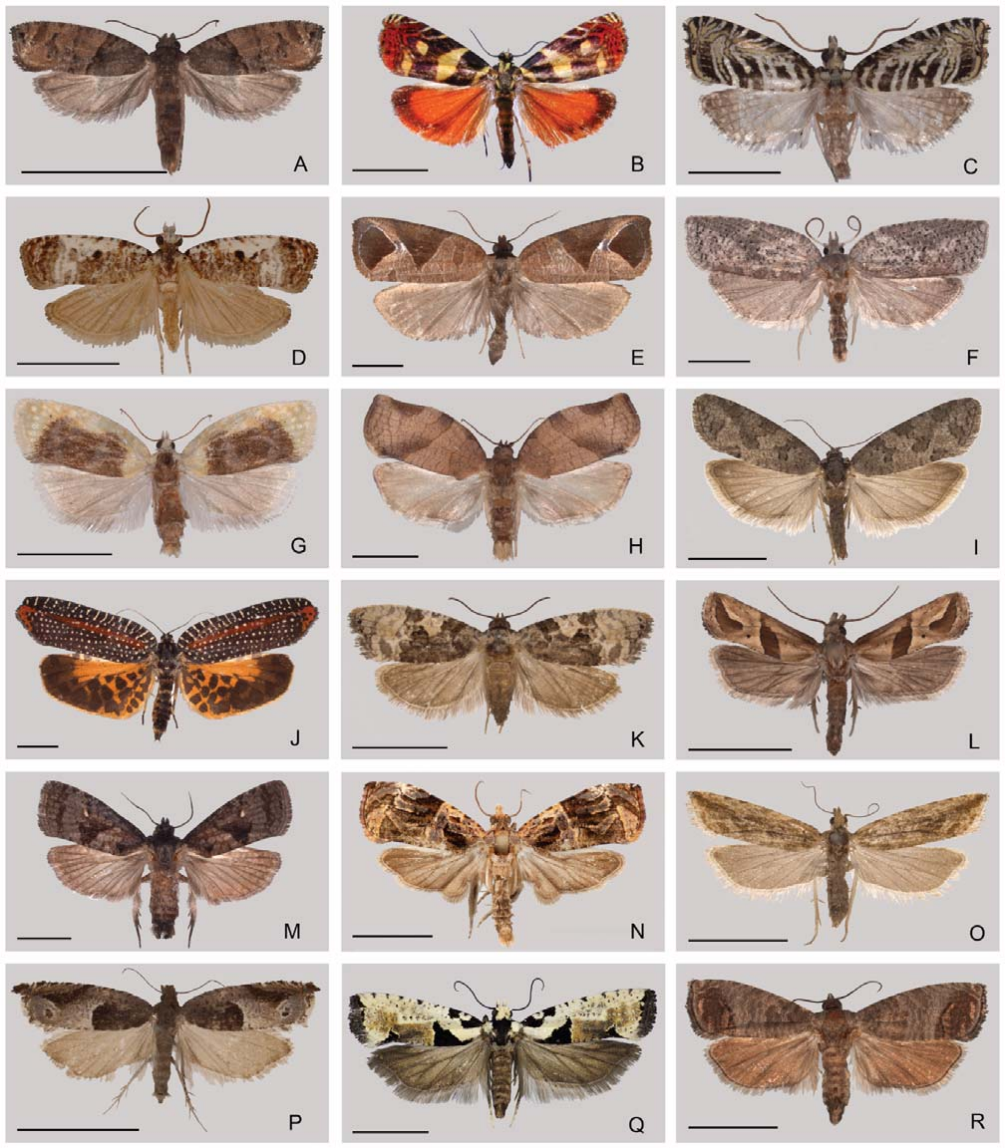
Adult habitus of representatives of Tortricidae used in the analysis. A–L, Tortricinae; M–R, Olethreutinae. A: Polyorthini, *Histura perseavora* Brown, Guatemala; B: Hilarographini, *Hilarographa* sp., Costa Rica; C: Chlidanothini, *Auratonota* sp., Costa Rica; D: Phricanthini, *Phricanthes asperana* Meyrick, Australia; E: Atterini, *Anacrusis stapiana* Felder & Rogenhofer, Costa Rica; F: Sparganothini, *Amorbia humerosana* Clemens, USA; G: Tortricini, *Acleris semipurpurana* Kearfott, USA; H: Archipini, *Choristoneura rosaceana* Harris, USA; I: Cnephasiini, *Cnephasia alfacarana* Razowski, Spain; J: Ceracini, *Cerace* sp., Japan; K: Euliini, *Bonagota* sp., Uruguay; L: Cochylini, *Carolella sartana* Hübner, USA; M: Microcorsini, *Cryptaspasma bipenicilla* Brown & Brown, USA; N: Olethreutini, *Afroploce karsholti* Aarvik, Tanzania; O: Bactrini, *Bactra furfurana* Haworth, Denmark; P: Enarmoniini, *Ancylis sparulana* Staudinger, Spain; Q: Eucosmini, *Gypsonoma paradelta* Meyrick, Tanzania; R: Grapholitini, *Cydia pomonella* Linnaeus, Spain. (A,C, E–H, J–M, US National Museum of Natural History, Smithsonian Institution; B, School of Biology, University of Costa Rica; D, Natural History Museum London; I, O, P, R, Cavanilles Institute of Biodiversity and Evolutionary Biology, University of Valencia; N, Q, Natural History Museum, University of Oslo). Scale bars: 5 mm.

The central goal of this study was to estimate relationships among the tribes and subfamilies of Tortricidae. We therefore sought to include representatives of as many of these as possible. The distribution of the 52 tortricid species we sequenced across the classification of Horak and R. Brown [24] is depicted in Table 3. We use this classification as the hypothesis to be tested because it is the most finely subdivided among recent schemes, permitting independent assessment of subsequent proposals for merging of tribes. Our sample encompasses all three subfamilies and 19 of the 22 tribes recognized by Horak and R. Brown [24]. Adult habitus images for representative species of all tribes are shown in Figure 2. We were unable to obtain fresh material for two of the eleven tribes of Tortricinae, the Oriental/Australian Schoenoteniini (17 genera, 204 species) and Epitymbiini (13 genera, 103 species), and for the small Oriental tribe Gatesclarkeanini (4 genera, 23 species) of Olethreutinae. Eleven tribes were represented by two or more genera, and the six largest tribes (670 to 1650+ species) were represented by three to six genera each. Outgroup choice was not straightforward because the phylogenetic position of Tortricidae among the lower ditrysians is quite unclear. We therefore included fourteen diverse outgroup taxa, listed in Table 4, representing all superfamilies which have been proposed as near relatives to Tortricidae by previous authors and/or previous molecular phylogenetic studies of ditrysian relationships [30]–[32], including preliminary analyses of the 800+ taxon Leptree project data set, which is described at http://www.leptree.net/status_matrix. The root of the entire tree, ingroups plus outgroups, was provisionally placed at Urodidae+Millieriidae, also on the basis of those preliminary analyses. Nomenclature for the outgroup taxa follows van Nieukerken et al. [34].

**Table 3:** Species sampled and their distribution across the current classification. Diversity numbers based on Baixeras et al. [2], distributions largely based on Horak [27], [29].

**CHLIDANOTINAE** (44 genera, 288 species)
**Polyorthini** (21 genera, 144 species; mainly Neotropical and Oriental/Australian): *Pseudatteria volcanica* (Butler), *Histura perseavora* Brown
**Chlidanotini** (18 genera, 76 species; Neotropical, Oriental/Australian): *Auratonota dispersa* Brown, *Heppnerographa tricesimana* (Zeller)
**Hilarographini** (5 genera, 68 species; mainly pantropical): *Hilarographa* sp.
**TORTRICINAE** (439 genera, 4,176 species)
**Phricanthini** (3 genera, 21 species; Australia, Southeast Asia, Madagascar): *Phricanthes asperana* Meyrick
**Atteriini** (8 genera, 45 species; mainly Neotropical): *Anacrusis nephrodes* (Walsingham)
**Sparganothini** (17 genera, 219 species; mainly New World): *Amorbia humerosana* Clemens, *Sparganothis reticulatana* (Clemens), *Platynota idaeusalis* (Walker)
**Ceracini** (4 genera, 29 species; eastern Palearctic, Oriental): *Cerace* sp.
**Archipini** (160 genera, 1,623 species; cosmopolitan): *Dichelia cosmopis* (Lower), *Atelodora* sp., *Clepsis melaleucana* (Walker), *Argyrotaenia alisellana* (Robinson), *Choristoneura rosaceana* (Harris), *Pandemis limitata* (Robinson)
**Cnephasiini** (19 genera, 261 species; mainly Himalayan; Oriental, Palearctic): *Decodes asapheus* Powell, *Cnephasia alfacarana* Razowski
**Tortricini** (41 genera, 406 species; nearly world-wide): *Acleris semipurpurana* (Kearfott), *Acleris affinatana* (Snellen)
**Euliini** (87 genera, 670 species; mainly Neotropical): *Bonogota* sp., *Eulia ministrana* L., *Pseudomeritastis* sp., *Netechma* sp.
**Cochylini** (75 genera, 1,028 species; cosmopolitan): *Aethes promptana* (Robinson), *Eugnosta busckana* (Comstock), *Carolella sartana* (Hübner)
**OLETHREUTINAE** (355 genera, 4,417 species)
**Microcorsini** (2 genera, 36 species; southern Hemisphere and Oriental): *Cryptaspasma querula* (Meyrick), *Cryptaspasma* sp.
**Bactrini** (9 genera, 113 species; cosmopolitan, mainly Old World): *Bactra furfurana* (Haworth), *Bactra maiorana* Heinrich
**Endotheniini** (5 genera, 52 species; cosmopolitan, mostly Holarctic): *Endothenia hebesana* (W.)
**Olethreutini** (133 genera, 1,077 species; cosmopolitan): *Oxysemaphora* sp., *Episimus tyrius* Heinrich, *Lobesia aeolopa* Meyrick, *Hedya dimidiana* (Clerck), *Olethreutes fasciatana* (Clemens), *Afroploce karsholti* Aarvik
**Enarmoniini** (38 genera, 298 species; nearly cosmopolitan, especially Oriental and Australian): *Ancylis sparulana* (Staudinger)
**Eucosmini** (106 genera, 1,651 species; cosmopolitan, predominantly Holarctic): *Spilonota eremitana* Moriuti, *Gypsonoma paradelta* (Meyrick), *Epinotia* sp., *Pelochrista zomonana* (Kearfott), *Epiblema abruptana* Walsingham, *Epiblema foenella* L.
**Grapholitini** (62 genera, 898 species; cosmopolitan): *Dichrorampha cancellatana* (Kennel) *Cryptophlebia illepida* (Butler), *Grapholita packardi* Zeller, *Grapholita delineana* Walker, *Cydia pomonella* (L.), *Multiquaestia purana* Karisch

**Table 4:** Outgroup species sampled.

**[Superfamily and family unassigned]:** *Heliocosma melanotypa* Turner
**Choreutoidea: Choreutidae:** *Brenthia sp.*
**Sesioidea: Sesiidae:** *Vitacea polistiformis* (Harris)
**Hyblaeoidea: Hyblaeidae:** *Hyblaea ibidias* Turner
**Zygaenoidea: Lacturidae:** [*unidentified*]
**Zygaenoidea: Limacodidae:** *Pantoctenia prasina* (Butler)
**Cossoidea: Cossidae:** *Archaeoses pentasema* (Lower)
**Gracillarioidea: Douglasiidae:** *Klimeschia transversella* Zeller
**Epermenioidea: Epermeniidae:** *Epermenia chaerophyllella* (Goeze)
**Galacticoidea: Galacticidae:** *Homadaula anisocentra* Meyrick
**Immoidea: Immidae:** *Imma tetrascia* Meyrick
**Copromorphoidea: Carposinidae:** *Sosineura mimica* (Lower)
**Urodoidea: Urodidae:** *Urodus decens* Meyrick
**[Superfamily unassigned]: Millieriidae:** *Millieria dolosalis* (Heydenreich)

The specimens used in this study, listed in Table S1, were obtained with the kind help of collectors around the world (see Acknowledgments). They are stored in 100% ethanol at −85°C as part of the ATOLep collection at the University of Maryland (details at http://www.leptree.net/collection). No permits were required for collection of these specimens, and no endangered species were used. DNA extraction used only the head and thorax for most species, leaving the rest of the body including the genitalia as a voucher, although the entire specimen was used for smaller species. Wing voucher images for most of our exemplars are posted at http://www.leptree.net/voucher_image_list, and DNA ‘barcodes’ for nearly all specimens have been kindly generated by the All-Leps Barcode of Life project http://www.lepbarcoding.org/. These data allow checks of our identifications against the BOLD (Barcode of Life Data system) reference library, and will facilitate future identifications of specimens whose identities are still pending (i.e., species listed as ‘sp.’ or ‘unidentified’ in this report).

The gene sample for this study, consisting entirely of protein-coding regions of nuclear genes, comprises two components. First, all taxa were sequenced for the five gene fragments described by Regier et al. [30], which total 6633 base pairs (bp), not including 333 with uncertain alignments. These genes are: *CAD* (2928 bp) [35], *DDC* (1281 bp) [36], *enolase* (1134 bp) [37], *period* (888 bp) [38] and *wingless* (402 bp) [39]. This set of genes has been used to resolve lepidopteran relationships at a variety of levels [30], [40]–[42].

To increase resolving power, all of the outgroup taxa, plus approximately half of the tortricids (29/52 = 56%), spread over all 19 tribes represented, were also sequenced for an additional 14 gene regions totaling 8193 bp. The 14 additional gene regions are a subset of the 21 new gene regions first tested across ditrysian Lepidoptera by Zwick et al. [43] and Cho et al. [32]. Based on the results of those studies, we selected these 14 gene regions as especially useful for further studies of Lepidoptera, as judged from their frequency of amplification and sequencing success and their total contribution to branch lengths. As detailed in Cho et al. [32], the 14 additional regions belong in turn to a group of 68 gene segments for which primers were first developed, and phylogenetic informativeness tested, across all the classes of Arthropoda [44] in a screen that included three diverse Lepidoptera, namely, *Prodoxus quinquepunctellus* (Prodoxidae, a non-ditrysian family), *Cydia pomonella* (Tortricidae, non-obtectomeran Apoditrysia) and *Antheraea paukstadtorum* (Saturniidae, Macroheterocera). GenBank numbers for the sequences are listed in Table S1, which also shows the total amount of sequence obtained for each exemplar.

### Generation of DNA sequence data

A detailed protocol of all laboratory procedures is provided by Regier et al. [44]. Further descriptions, including gene amplification strategies, PCR primer sequences, and sequence assembly and alignment methods, can be found in Regier et al. [30], [38], [40], [44]. To summarize, total nucleic acids were isolated and specific regions of the cognate mRNAs were amplified by RT-PCR. Specific bands were gel-isolated and re-amplified by PCR using hemi-nested primers, when available. Visible bands that were too faint to sequence were re-amplified using the M13 sequences at the 5′ ends of all primers. PCR amplicons were sequenced directly on a 3730 DNA Analyzer (Applied Biosystems). Sequences were edited and assembled using the TREV, PREGAP4 and GAP4 programs in the STADEN package [45]. Multi-sequence alignments were made using the Translation Align program within the Geneious Pro 5.3.4 software package (Protein alignment option: Geneious Alignment; Cost matrix: Blosum62; Gap open penalty: 12; Gap extension penalty: 3; Alignment type: Global alignment with free end gaps; + Build guide tree via alignment; Refinement iterations: 2). A data-exclusion mask of 1440 characters out of 20,373 total aligned characters ( = 7.1% of total) for all 66 species was applied.

### Character partitions, taxon×gene data set design, and phylogenetic analyses

Previous studies using these same genes [30], [32] have shown that in some regions of the phylogeny of Lepidoptera, sites undergoing synonymous substitutions are prone to among-lineage base compositional heterogeneity, thereby obscuring and sometimes misleading phylogeny inference. For this reason, in addition to using all data unpartitioned, we performed analyses in which the majority of synonymous change was either independently modeled or excluded. To independently model synonymous change, we partitioned characters into sets undergoing mostly synonymous versus mostly non-synonymous change following Regier et al. [30]. We first isolated the subset of sites at the first codon position (nt1) which encode no leucine or arginine residues in any species in the data set, using a Perl script available online (Appendix 4 of [40]; see http://www.phylotools.com for most recent version). Since only leucine and arginine codons can undergo synonymous change at nt1, synonymous change is not directly detectable in any pairwise comparisons of extant taxa for such characters. We combined these sites with nt2 to produce a partition, termed “non-synonymous nt1+nt2”, which should reflect only non-synonymous change (identical to the “noLR1+nt2” of Regier et al. [40]). The excluded sites were then combined with nt3 to create a partition termed “potentially synonymous nt1+nt3.” The great majority of changes in this partition should be synonymous, though there will also be a few non-synonymous substitutions at both nt1 and nt3. The purpose of this partitioning scheme is to improve separation of non-synonymous from synonymous change over that achieved by partitions based solely on codon position.

If compositional heterogeneity is strong, even partitioning may not overcome its deleterious effects on phylogenetic inference, since partitioning itself does not correct for heterogeneity but instead can reduce its influence only indirectly, through “down-weighting” of the rapidly evolving (and compositionally heterogeneous) synonymous change. For this reason, we also used “degen1” coding [46], which eliminates the contribution of any synonymous change to pairwise differences between extant taxa (see http://www.phylotools.com for Perl script). Degen1 is an extension of the RY coding scheme [47]. Nucleotide sites at any codon position that have the potential of directly undergoing synonymous change, by virtue of the specific codon they are part of, are fully degenerated, using standard IUPAC codenames. For example, CAC and CAT (His) are both coded CAY, while TTA, TTG, CTT, CTC, CTA and CTG (Leu) are all coded YTN. Synonymous change becomes largely invisible to phylogenetic inference methods, and any compositional heterogeneity it produces is eliminated. The substitution model used in all analyses was GTR+gamma+I. This model was applied separately to each character subset in the partitioned analysis.

Our somewhat unconventional sampling plan, in which only about half the ingroup taxa were sequenced for the full set of 19 genes, was designed to maximize efficiency of resource use in resolving both deeper and shallower nodes within Tortricidae. The effectiveness of such deliberately incomplete gene sampling, which in theory might be undercut by phylogenetic artifacts resulting from the large blocks of missing data [48], [49], has been supported by simulations [50] and by a growing body of case studies ([32], [51], [52] and references therein). To ensure that our results are not subject to artifacts from deliberate blocks of missing data, and to add to the empirical evidence on this issue, we carried out parallel analyses on the full, deliberately incomplete 19 gene data set and on a reduced gene sample, the “five-gene complete matrix,” comprising only the five gene regions sequenced in all 66 taxa. If the large blocks of missing data created by our design result in artifactual groupings, we might expect to see strong support, in trees from the expanded, deliberately incomplete matrix, for groups which do not occur in trees from the five-genes-only matrix [32]. Conversely, finding the same topology from the two matrices would imply that large missing data blocks in the full deliberately incomplete matrix do not themselves mislead phylogenetic inference. Even if it did not induce artifacts, however, deliberately incomplete augmentation of gene sampling would be an ineffective strategy if it failed to strengthen phylogenetic signal, or worse, obscured it. Therefore, we also asked whether bootstrap support was increased or decreased, on average and for which and how many nodes, by the deliberately incomplete 19-gene matrix as compared to the five-gene complete matrix.

All of our phylogenetic analyses were based on the maximum likelihood criterion as implemented in GARLI (Genetic Algorithm for Rapid Likelihood Inference; version 1.0) [53]. We used the program default settings, including random stepwise addition starting trees, except that we halved the number of successive generations yielding no improvement in likelihood score that prompts termination (genthreshfortopoterm = 10000), as suggested for bootstrapping in the GARLI manual. Each search for an optimal tree consisted of 999 to 1090 GARLI runs, while bootstrap analyses consisted of 742–901 pseudo-replicates, each based on 15 heuristic search replicates. Optimal-tree searches and bootstrap analyses were parallelized using Grid computing [54] through The Lattice Project [55]. For consistency in the characterization of results, we will refer to bootstrap support of 70–79% as “moderate” and support ≥80% as “strong.” We emphasize that these terms are for heuristic purposes only. Phylogenetic trends in host plant use

To examine postulated trends in the evolution of tortricid larval feeding habits [34], we compiled a synopsis of host use traits for each tribe, using primarily the data base of J. Brown et al. [56], supplemented by Janzen and Hallwachs [57] and other sources as noted below. For each tribe we scored predominant feeding mode/location (e.g., leaf roller, stem borer, etc.), predominant host families used (if any), host range ( = diet breath), and pattern of oviposition (eggs laid singly, in small groups or in large clusters). The latter has been hypothesized to correlate with host range. Host range itself is highly variable and difficult to define and measure. To capture possible broad-scale evolutionary trends, we used a heuristic definition and procedure, recognizing just two categories, oligophagy and polyphagy We define oligophagous species to be those that use only, or almost only, hosts in a single plant order as defined by APG III [58], while polyphagous species are those that use hosts in two or more plant orders. The J. Brown et al. database [56], from which we extracted relative numbers of oligophagous versus polyphagous species in each tribe, is a spreadsheet that attempts to incorporate all known observations of tortricid larval host associations. Each of the 10,000+ entries represents the association of a single tortricid species with a particular plant species, attested to by one or more reports (which may or may not be independent. More than one entry can be present for the same tortricid/plant association if there is good reason to believe that the additional sets of observations are independent of the first, e.g., if they represent a different geographic region. There are always multiple entries for a tortricid species if it is reported from more than one plant species. Our tallies of oligophagous and polyphagous species excluded all species represented by a single entry in the table. We reasoned that to include species for which there may be only a single independent observation on feeding habits, on a single plant species, could artificially inflate the apparent number of oligophages. Of course, this criterion may also cause us to disregard species that truly are oligophagous. Tortricid species making up multiple entries in the data base were scored as oligophagous if ≥80% of those entries represented associations with the same plant order; otherwise, they were scored as polyphagous. The 80% criterion is an attempt to avoid under-counting oligophagous species due to erroneous records or occasional records from rarely-used, suboptimal hosts, a recognized source of error in other food plant data bases [59]. Because of the many sources of possible error, our compilation is useful mainly as a comparative rather than absolute measure of % polyphagous species.

## Results

**Figure 3:**
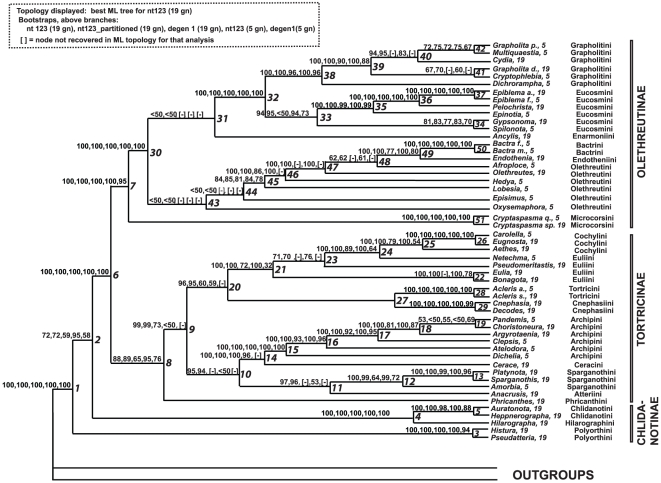
Maximum likelihood estimate of phylogenetic relationships in Tortricidae. Tree was obtained from 1000 GARLI searches under a GTR+gamma+I model for all nucleotides (unpartitioned). Bootstrap support values (742–901 pseudoreplicates) above branches for: nt123 (19 genes), nt123_partitioned (19 genes), degen1 (19 genes), nt123 (5 genes), degen1 (5 genes). “[-]” = node not present on best ML tree for that analysis. Nodes within Tortricidae are numbered (to the right of node) for purposes of discussion.

**Figure 4:**
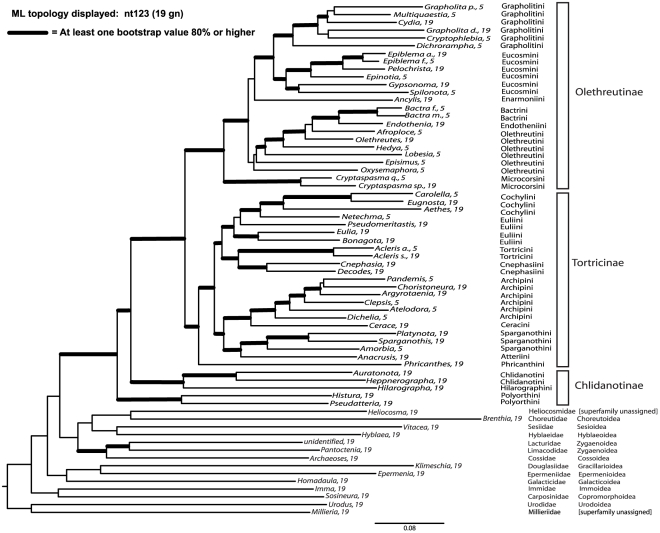
Phylogram presentation of best maximum likelihood tree obtained from 1000 GARLI searches. Model used was a GTR+gamma+I model for all nucleotides (unpartitioned). Thickened branches are supported by ≥80% bootstrap in at least one analysis (see Fig. 3).

Figures 3 and 4 show the best ML tree found in 1000 GARLI searches using nt123 (unpartitioned) for the 19-gene deliberately incomplete data set, with bootstrap values for all analyses superimposed on the branches in Figure 3. The nodes are numbered to facilitate presentation. Within Tortricidae, we find topologies that are strongly supported at all levels of divergence and that differ little among data sets and character treatments. For the 19-gene deliberately incomplete data set, using either nt123 or nt123 partitioned, the fractions of nodes (51 total) with bootstrap support of ≥70%, ≥80% and ≥90% were 90%, 82% and 76% respectively. Of the five few nodes showing weak bootstrap support in all analyses (<70%), four involve deeper relationships among tribes of Olethreutinae, clearly the most problematic region of the tree.

The strongest overall support in our analyses comes from the nt123 and nt123 partitioned character treatments. However, many nodes are also strongly supported by the non-synonymous-only ( = degen1) data set (67%, 55% and 45% of nodes, respectively, with bootstrap values of ≥70%, ≥80% and ≥90%). While the non-synonymous-only data set yielded a few nodes that conflict with the nt123 tree, these nodes were only weakly supported (bootstraps ≤51%). Thus, there is little sign of conflict in apparent signal between synonymous and non-synonymous change (but see below). There are also no suggestions of phylogenetic artifacts arising from the large blocks of missing data in the 19-gene deliberately incomplete matrix, as this matrix and the five-gene complete matrix give essentially identical topologies except for nodes which are very weakly supported in all analyses. The five-gene matrix alone is highly informative about relationships within tortricids, providing bootstrap support comparable to that from the 19-gene matrix for most nodes (even higher in a few cases, e.g., **node 2**). For three nodes embodying deeper divergences within Tortricinae, however, substantial support is evident only when all 19 genes are included.

Support for several nodes was greatly increased by the additional 14 genes, despite the fact that these were sequenced in only about half of the taxa (29/52), and there was no indication of phylogenetic artifacts from the deliberately incomplete gene sampling design. Our results thus provide further support for the effectiveness of augmenting the gene sample in only a subset of taxa, as a resource-efficient approach to improving node support [32].

In contrast to relationships within Tortricidae, relationships among the outgroup taxa are highly unstable to differences among data matrices and character treatments, and strong bootstrap support is almost entirely lacking.

## Discussion

**Figure 5:**
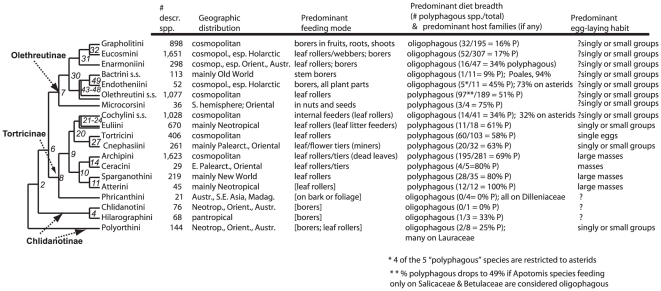
Synopsis of species diversity, distribution and larval host-plant use for the tribes of Tortricidae studied, mapped onto a phylogeny condensed from **Fig. 3.** The multiple branches leading to the names Eucosmini and Olethreutini s.s. denote the paraphyly of these tribes discovered here. Col. 1 = number of described species, taken from Table 3. Col. 2 = summary of geographic distributions, following Horak [29]. Cols. 3, 4, 5 = summary statements of predominant mode of larval feeding, host range, host taxa used and egg-laying habits, following Horak [29], Powell et al. [33] and J. Brown et al. [56]. ‘[]’ denotes assertion based on very few observations; preceding question mark denotes assertion based on uncertain or conflicting information; ‘( )’ denotes habits significantly represented but markedly less common than alternative; ‘;’ separates habits of similar frequency. In column 4, “oligophagous” = feeding on a single plant family, “polyphagous” = feeding on two or more plant families, as described in the text. Numbers in parentheses, compiled from Brown et al. [56], are number of polyphagous species/total number of species with two or more foodplant observations = % polyphagous species.

In this section we first review the agreement and disagreement of our molecular results with previous hypotheses on the phylogeny of Tortricoidea. Finding strong evidence for paraphyly of two tribes, Euliini and Olethreutini, we propose formal taxonomic changes for both. A summary of our findings regarding monophyly or lack thereof for each of the tribes and subfamilies of Horak and R. Brown [24] is provided in Table 2. The information on diversity, distribution and life history given in the accounts for individual tribes is summarized in Figure 5. We end with an overview of the new phylogeny's bearing on previous postulates about evolutionary trends in tortricid larval feeding habits. In addition, we provide an illustrated on-line synopsis of each tribe as currently understood at http://www.leptree.net/lep_taxon_page/Tortricidae/view.

### Phylogenetic position of Tortricidae and basal divergences within the family

Like previous molecular studies [30]–[32], the present results provide essentially no credible support for any hypothesis about the sister group to Tortricoidea, despite the availability of 19 genes of sequence (up to 14.8 kb) for all 14 outgroup taxa (see Fig. 4). For example, the tortricid-like genus *Heliocosma*, sometimes included in Tortricoidea but excluded by Horak et al. [60], never groups next to Tortricidae. Relationships among the superfamilies of lower apoditrysian Lepidoptera appear to be a very difficult phylogenetic problem [32].

Our results do however provide very clear resolution for a majority of relationships within Tortricidae, as evidenced in Figures 3 and 4. Monophyly for the family (Fig. 3, **node 1**) receives 100% bootstrap support in all analyses, consistent with traditional morphological concepts as well as recent molecular analyses [30]–[32]. Basal divergences within the family are also mostly strongly supported. The monophyly of Olethreutinae [24], strongly corroborated here (**node 7**; bootstrap percentage [BP] = 100%), is supported by morphological characters including a single ring of scales on the antennal flagellomeres and fusion of the phallus to the juxta through the anellus.

Somewhat more surprisingly, monophyly for Tortricinae (22 genera, 9 of 11 tribes sampled) is supported almost as strongly (**node 8**; 88% bootstrap for nt123, 19 genes). The ML analysis of Mutanen et al. [31] also recovered a monophyletic Tortricinae, based on a smaller, mostly non-overlapping sample (nine genera), but with <50% bootstrap support, probably because they excluded nt3. For the past two decades, Tortricinae have been thought by most tortricid workers to be para- or polyphyletic [24], [29], because convincing morphological synapomorphies have not been found, although no one proposed constituent taxa whose removal would render it monophyletic. Our results strongly suggest that the search for tortricine synapomorphies deserves more effort.

Our analyses unambiguously support a sister group relationship between Tortricinae and Olethreutinae (**node 6**; BP = 100), confirming the prevailing view of Chlidanotinae as the earliest-diverging subfamily [24], [61]. The same hypothesis is strongly supported by the results of Mutanen et al. [31], based on a smaller taxon sample.

The more problematic question about basal tortricid relationships is the monophyly of Chlidanotinae. In all the analyses for this study, Chlidanotinae were inferred to be paraphyletic, with Chlidanotini+Hilarographini allied to the remaining subfamilies to the exclusion of Polyorthini (**node 2**; BP = 79 for nt123 partitioned).). However, in ongoing studies of much larger data sets (>400 outgroups to Tortricidae, across all Lepidoptera; data not shown), analyses that included synonymous change (nt123, nt123 partitioned) favored monophyly for Chlidanotinae with bootstrap support up to 77%, while paraphyly was almost always favored when synonymous change was excluded (i.e., degen-1 analysis), albeit with bootstrap <50%. Seeking the reasons for this unusual conflict, we experimented with widely differing outgroup samples. We found, surprisingly, that the divergent results from nt123 can be ascribed entirely to base composition heterogeneity, among the outgroups and between these and Tortricidae. That is, outgroups with some patterns of composition yield paraphyly for Chlidanotinae, while those with other patterns, no more or less closely related to Tortricidae, yield monophyly. Thus, synonymous change seems to provide no reliable information regarding chlidanotine monophyly. Non-synonymous change, in contrast, consistently favors paraphyly, which we therefore take as our working hypothesis, although bootstrap support is not strong. However, this conclusion contradicts the widely accepted view that Chlidanotinae are monophyletic, which is supported most clearly by a complex morphological synapomorphy: in all chlidanotines and in no other Lepidoptera, the valve of the male genitalia bears a deep longitudinal invagination that holds distinctive hairpencils arising from the eighth abdominal segment [12], [16], [24]. On the molecular tree, one must interpret this suite of traits as either arising at the base of Tortricidae and undergoing subsequent loss, or as arising independently in Polyorthini and in Chlidanotini+Hilarographini.

The question of subfamily monophyly aside, the relative positions of the three tribes of Chlidanotinae conform to those proposed by previous authors based on morphology [16], [24], [62]–[64], including Horak [29], who suggested that Hilarographini may be subordinate within Chlidanotini. Our two exemplar genera of Polyorthini (Fig. 2A) group strongly with each other, as do the two exemplars of Chlidanotini (*Auratonota* [Fig. 2C] and *Heppnerographa*); and, the latter in turn group strongly with the representative of Hilarographini (Fig. 2B), to the exclusion of Polyorthini. In our trees, therefore, Polyorthini are the earliest-branching group of Tortricidae. All three tribes of Chlidanotinae are primarily tropical. Polyorthini comprise about 144 species found mainly in the Neotropical and Oriental/Australian regions, a distribution similar to that of Chlidanotini, which include 76 species, whereas the 68 species of Hilarographini are pantropical. Life history observations on all three tribes are few, but suggest that all three are internal feeders or leaf rollers on living plants [29].

### Relationships within Tortricinae

When all 19 genes are included, our data provide especially clear resolution of relationships among and within the nine (of 11) sampled tribes of Tortricinae. There is very strong support for placement of Phricanthini (Fig. 2D) as sister group to the remaining Tortricinae (**node 9**; BP = 99). The subfamilial assignment of Phricanthini has been in question owing to the many distinctive features that this tribe shares with few or no other tortricids, such as an upright (lemon-shaped) egg, abdominal tergal spines, presence of a well-developed saccus in the male genitalia (in some but not all species), and enlarged SD pinacula on the larva [65]–[68]. Phricanthini are also unusual in that the larvae are restricted to the early-diverging eudicot family Dilleniaceae, where they feed either in shelters on foliage or on living bark [29]. While these features have been used to argue for placement of Phricanthini at the base of the entire family, they now appear to represent autapomorphies rather than symplesiomorphies with outgroup families. Phricanthini comprise 21 species found in Australia, Southeast Asia and Madagascar. Their presence in continental Africa and in the Neotropics is almost certainly the result of inadvertent introductions [69].

The remaining Tortricinae diverge basally into two clades, one of which consists of Archipini+Ceracini and Sparganothini+Atteriini (**node 10**; BP = 95). Members of this clade are characterized by slender, spine-like, deciduous cornuti on the vesica of the phallus. The clade is further corroborated by a multi-step transformation series in egg-laying behavior proposed by Powell [20] (see our Fig. 1A). The condition of laying single eggs or small clusters, shared by all other Tortricinae in our sample, was considered primitive by Powell [20]. In contrast, females in Archipini, Ceracini, Sparganothini, and Atteriini lay their eggs in large clusters, over which the female deposits a layer of colleterial secretion. In an additional step, females of Atteriini have have specialized scales on the venter of the abdomen that are used to build a “fence” around the large egg mass covered by colleterial secretion [68], [70]. The grouping of Atteriini with Sparganothini, strongly supported here (**node 11**; BP = 99), is further suggested by morphological traits that have often been interpreted as symplesiomorphies rather than synapomorphies, such as the male genitalia having large, rectangular, simple valva and densely scaled socii. Atteriini (Fig. 2E) include 45 species, mainly Neotropical, while Sparganothini (Fig. 2F), also mainly New World, comprise 219 species. The larvae in both tribes are mostly polyphagous leaf rollers [29], [33], [57].

The strongly supported grouping (**node 14**; BP = 100) of Archipini with Ceracini (Fig. 2J; considered a distinct family by Diakonoff [71]) was suggested by several previous authors [22]–[24] based on many morphological similarities, in particular, the overall configuration of the male genitalia. Monophyly for Archipini as sampled here is also strongly supported, as in previous molecular analyses [30], [31], but no definite synapomorphies are known. A large number of genera are unambiguously assigned to the tribe based on a brush of hairs from the venter of the uncus in the male genitalia and a curved, spine-shaped signum with a capitulum in the female genitalia, and this core of genera includes *Choristoneura* (Fig. 2H), *Archips*, *Pandemis*, *Argrotaenia*, *Clepsis*, *Epiphyas* and many others. However, extensive additional taxon sampling might reveal a slightly different picture, as the tribe also includes some provisionally assigned genera (e.g., *Pseudargyrotoza*, *Mictopsichia*) that do not fit convincingly based on morphological features. Also absent from our study are the Ramapesiini sensu Razowski [72], [73], usually considered a subordinate group within Archipini [1], [29]. The included genera (e.g., *Gnorismoneura*, *Egopepa*, *Batodes*, *Aneuxanthis*, *Geopepa*, *Epagoge*, *Ramapesia*) are characterized by a valva in the male genitalia with a more strongly sclerotized costa than is typical in Archipini. According to the morphology-based analysis of Jinbo [74], Ramapesiini comprise an assemblage of plesiomorphic genera that may or not be monophyletic and sister group to the remaining Archipini.

Archipini and Ceracini differ dramatically in diversity. The former are a cosmopolitan group of 1,623 described species. The latter, which bear multiple conspicuous synapomorphies (e.g., large, day-flying adults with brightly colored forewings, female frenulum with 4 or more acanthi, loss of dorsal spines on the pupa), include only 29 species restricted to the eastern Palearctic and Oriental regions. Both tribes are leaf roller/tiers, with a strong tendency to polyphagy [29], [33].

The second large clade of Tortricinae minus Phricanthini consists of Tortricini+Cnephasiini and Euliini+Cochylini (**node 20**; BP = 96). Members of this clade typically have non-deciduous cornuti on the vesica of the phallus (i.e., Tortricini, Cochylini, and some Euliini) or lack cornuti altogether (i.e., Cnephasiini). The grouping of Euliini (Fig. 2K) with Cochylini (Fig. 2L), very strongly supported here (**node 21**; BP = 100), was also recovered by Mutanen et al. [31], and accords with the view of Kuznetsov and Stekolnikov [17], [22] based on musculature of the male genitalia (see Figs. 1B, 1F).

Monophyly of Cochylini as sampled here is also strongly upheld (**node 25**; BP = 100). Morphological synapomorphies of Cochylini include: in the male genitalia, loss of the gnathos and membranous medial connection of the arms of the vinculum; in the female genitalia, usually with an accessory bursa, frequently lacking differentiation between the corpus bursae and the ductus bursae, and corpus bursae with complex sclerites, but never with a well-defined signum; in the forewing, a deflexed apical portion when in typical resting posture, vein CuP strongly reduced or absent and vein CuA2 originating from the distal two-thirds of the discal cell; and in the larva, an enlarged L-pinaculum on T1, extending posteriorly beneath the spiracle, as well a bisetose L-group on segment A9. In addition, the uncus of the male genitalia is absent, the associated depressor muscles (M1) are reduced, the male forewing lacks a costal fold, and the female frenulum consists of two acanthi in all but the earliest-diverging Cochylini (i.e., *Phtheochroa*, *Henricus*) [75], [76]. Cochylini are a cosmopolitan group of about 1000 species. The larvae are usually oligophagous, feeding internally in plant reproductive parts, stems, or roots, often on Asteraceae [56], [77].

In contrast, there is very strong evidence against the monophyly of Euliini: as currently defined the tribe is paraphyletic with respect to Cochylini (**node 24**; BP = 100). The definition of Euliini has long been problematic. The group was proposed as a subtribe of Cochylini by Kuznetsov and Stekolnikov [17] based on the Holarctic genus *Eulia*, but was subsequently elevated to tribal status by Powell [78] and redefined to consist almost entirely of Neotropical genera. Many of the included genera were initially described in Archipini by Razowski [79]. The tribe has at times been considered an assemblage of similarly plesiomorphic tortricines [80], [81]. The presence of a unique male foreleg hairpencil was hypothesized to represent a synapomorphy for Euliini by J. Brown [80], but this structure subsequently was found in at least one cochyline genus as well (*Aethes*; JWB, unpublished observations). Hence, although the character is highly variable (perhaps easily lost or suppressed), as are many male secondary sexual structures, it may represent a synapomorphy for Euliini+Cochylini. The same structure is widespread among males of Schoenotenini, which were not included in our analysis. As currently defined, the Euliini contain 670 species. The larvae are ecologically diverse, including polyphagous leafrollers [82], stem-borers [83], and leaf litter feeders [84].

The strong evidence for paraphyly of Euliini dictates that the tribe should either be subdivided into multiple monophyletic tribes, or combined with Cochylini to eliminate paraphyly. As an interim solution we choose the latter, and hereby formally synonymize Euliini with Cochylini. Our justification is as follows. The taxon sampling of the present study, designed to estimate relationships among tribes, provides very limited insight into the structure within them. Current understanding of relationships of the genera now assigned to Euliini is so incomplete that attempting to assign them to monophyletic tribes would leave many incertae sedis. Conversely, given the strong support for the clade Euliini+Cochylini, a broadened definition of Cochylini allows confident assignment of all current euliine genera to a monophyletic tribe. Continued studies may reveal an increasing number of monophyletic subgroups of former Euliini, for example one centered on *Eulia*+*Bonagota* (**node 22**; BP = 100), eventually allowing reclassification and resurrection of a family-group name based on *Eulia*. In the meantime, the clearly monophyletic Cochylini s.s. may be provisionally referred to as the subtribe Cochylina.

The strongly-supported grouping (**node 27**; BP = 100) of Cnephasiini (Fig. 2I) with Tortricini (Fig. 2G) contradicts the hypotheses of Kuznetsov and Stekolnikov [17], [21], [22] (see Figs. 1B, 1D, 1F) and Razowski [23], but conforms to the view of Powell [20] (Fig. 1A), who cited loss of the costal fold in the male forewing as a shared character change. The proposed relationship is also supported by other characters not previously recognized as synapomorphies, such as the distinctive stellate signum seen in the female genitalia of both tribes, and a “floricomous ovipositor” that is universal in Cnephasiini and also occurs sporadically in Tortricini. Powell & Common [68] suggested that these specialized setae of the papillae anales were “analogous” between the two tribes. The two tribes also share the condition of laying eggs singly or in small clusters and the lack of a male forewing costal fold (as mentioned above), but these are probably symplesiomorphies. Contrary to Powell [20], the tortricid ground plan is now thought to lack the costal fold, which is also missing in most outgroups and in basal tortricids (Chlidanotinae and Phricanthini). Moreover, the phylogenetic interpretation of male secondary sexual characters in Tortricidae has long been controversial [85], [86], and a male costal fold occurs sporadically throughout Olethreutinae and Tortricinae.

Monophyly for Cnephasiini as sampled here is strongly supported (**node 29**; BP = 100) bootstrap), but corresponding morphological synapomorphies are not obvious, because, as discussed above, some of the characters previously thought to support this tribe are now seen as linking them with Tortricini. The distinctive shape of the papillae anales, the finely spined transtilla, and the absence of cornuti are possible synapomorphies. However, circumscription of a monophyletic Cnephasiini is still a work in progress, as some taxa traditionally assigned there seem clearly not to fit, lacking even the characters uniting the tribe with Tortricini. An example is the Australian *Arotrophora* group (sensu Common [87]), restricted to Proteaceae, included in “Cnephasiini sensu lato” by Horak et al. [60] but later “excluded from the Cnephasiini” by Horak [29]. The tribe as currently delimited contains 261 species and is most diverse in the Oriental and Palearctic regions. The larvae typically feed on tied leaves or tunnel in flowers.

Monophyly of Tortricini was previously supported by the molecular study of Razowski et al. [88], who sampled 23 species in four genera. Morphological synapomorphies for Tortricini include the loss of the uncus, the development of a subscaphium, the development of a brachiola (a unique membranous digitate projection from the valva), raised scales on the forewing, and often highly polymorphic forewing patterns. In addition, adult diapause occurs in many members of this tribe and nowhere else in the family. The tribe is cosmopolitan and includes 406 described species. Larvae are typically leaf rollers, feeding collectively on a very wide range of host taxa and growth forms, but with individual species often oligophagous [33], [56].

### Relationships within Olethreutinae

Our data strongly support placement of Microcorsini as sister group to the remaining Olethreutinae as sampled here (**node 30**; BP = 100). The relatively basal position of Microcorsini is supported by their retention of some apparently plesiomorphic traits shared with Tortricinae. In the female genitalia the sterigma is connected to the apophyses anteriores, and in the male genitalia the coecum of the phallus is large (absent in the rest of Olethreutinae); in the pupa there is an alar furrow with raised margins [29]. Microcorsini (Fig. 2M) are a small, possibly relict group consisting of 36 species in two genera, found on all the southern continents with northward extensions in the Oriental region to Japan and in the Neotropical region to Mexico and the southeastern United States. The larvae bore in nuts, seeds and fruits [29], [89].

Two large subclades of Olethreutinae apart from Microcorsini are strongly supported. One of these consists of Eucosmini+Grapholitini (**node 31**; BP = 100%), a grouping proposed by previous authors including Razowski [23] (Fig. 1C) and Kuznetsov & Stekolnikov [17], [21], [22] (see Figs. 1B, 1D, 1E). A possible synapomorphy is that in the females of both tribes the sterigma is derived from a smooth periostial sclerite. Both tribes also bear a distinct speculum (ocellar area) on the upper side of the forewing, only faintly visible in a few Olethreutini. This is however a plastic character that may disappear or be strongly modified secondarily (as in *Dichrorampha*).

The molecular data strongly support monophyly of Grapholitini (**node 38**; BP = 100%), for which Komai [90] proposed as a possible apomorphy a shortened male sternum 8 with a straight posterior margin. However, the generic groupings within Grapholitini proposed by Danilevsky and Kuznetsov [91] and refined by Komai [90] are not exactly recovered by our data. Komai recognized a *Grapholita* group, a *Cydia* group, and a *Dichrorampha* group for the Palaearctic fauna; Komai & Horak [92] added a fourth group from Australia (*Loranthacydia* group, not represented in our sampling). The basal position of *Dichrorampha* is in agreement with Komai's view. *Grapholita* itself as currently defined, however, appears not to be monophyletic. One of the two species sampled here, *G.* (*Aspila*) *packardi*, groups strongly (**node 40**; BP = 95) with *Cydia* (Fig. 2R; in the “*Cydia* Genus Group” of Komai) and the newly-discovered *Multiquaestia*, as predicted by Aarvik and Karisch [93], whereas the other, *G. (s. str.) delineana*, groups with *Cryptophlebia* (**node 41**; BP = 70); both are in the “*Grapholita* Genus Group” of Komai. Horak and R. Brown ([24]: their figure 1.2.5) hypothesized Grapholitini to be a polyphyletic group, derived from within Olethreutini and Eucosmini and defined by convergent reductions in the male genitalia, i.e., loss of the uncus and socius, and hindwing venation with M_2_ distant and parallel to M_3_, a hypothesis later rejected by Horak [27]. Our analyses provide no support for this hypothesis. Grapholitini are a cosmopolitan group of 898 described species. The larvae are typically oligophagous borers in fruits, shoots or roots, collectively spanning a very wide range of host plant taxa and growth forms [29], [56].

Monophyly for Eucosmini as sampled here is also well supported by our data (**node 33**; BP = 94) in agreement with preexisting views [17], [21]–[23]. However, no morphological synapomorphy is known for the entire tribe, one of the largest in Tortricidae, and its precise limits are unclear. The classical character, hind wing vein M_2_ curved and approximated to the stalk of M_3_ and CuA_1_ [92], [94], is far from universal [27]. Other characters common in but not exclusive to Eucosmini were compiled by Horak [27]. Because we focused on relationships among tribes, we chose exemplars that were clearly assignable to Eucosmini (e.g., Fig. 2Q), avoiding taxa of uncertain affinity. Thus, our study does not address the question of the circumscription of Eucosmini. Eucosmini comprise 1,651 species, cosmopolitan but predominantly Holarctic in distribution. According to Horak [29], the larvae include both leaf rollers and webbers, and borers in roots, stems and fruit, with the latter habit characterizing more derived taxa.

Both the monophyly and position of Enarmoniini, here represented by *Ancylis* (Fig. 2P), have been controversial. Kuznetsov and Stekolnikov's proposal [22] of a ‘supertribe Eucosmidii’ including Enarmoniini, Eucosmini and Grapholitini (Figs. 1B, 1D, 1E) reflects the widespread view that Enarmoniini are closely related to Eucosmini. *Ancylis* is allied with Eucosmini in some of our analyses, but its position is weakly supported and varies among analyses (**node 13**; BP<50). However, Our data do strongly support removal of Enarmoniini from Eucosmini, where they were assigned historically [24], [95], [96], as well as from Eucosmini+Grapholitini. The tribe was recently redefined by Horak [27] to include taxa previously in Eucosmini, but no convincing evidence of its monophyly was found. Enarmoniini as presently delineated include 298 species, cosmopolitan in distribution, but especially diverse in the Oriental and Australian regions. The larvae include both leaf rollers and borers [29], typically with restricted host ranges [56].

The remaining subclade of Olethreutinae minus Microcorsini in our results consists of Bactrini (*Bactra*), Endotheniini (*Endothenia*) and Olethreutini (**node 43**). Apart from *Episimus* and *Oxysemaphora*, whose placements are weakly established (**nodes 43, 44**; BP<50), this clade is strongly supported (**node 45**; BP = 84). It corresponds approximately to the supra-tribal group “Olethreutidii” of Kuznetsov and Stekolnikov [17], [22] (Figs. 1D, 1E), adopted by Safonkin [25] (Fig. 1F), equivalent in turn to the broad concept of Olethreutini of Diakonoff [97]. Diakonoff divided Olethreutini s.l. into 12 subtribes, of which five are represented in our sampling: “Bactrae” (by *Bactra*); “Lobesiae” (by *Lobesia*), “Endotheniae” (by *Endothenia*), “Neopotamiae” (by *Afroploce*); and “Olethreutae” (by *Oxysemaphora*, *Hedya* and *Olethreutes*). Subsequent authors, including Kuznetsov and Stekolnikov (see Fig. 1), treated some of these groups as tribes.

Within the “Olethreutidii” clade (**node 43**), the tribes Bactrini and Endotheniini, each represented by its type genus, are strongly supported as sister groups (**node 49**; BP = 100). The monophyly of Bactrini, unquestioned since the tribe was proposed by Falkovitsh [84], is supported by multiple synapomorphies, including a spined globular sacculus in the male genitalia, an anteriorly invaginated sterigma in the female, and a characteristic cryptic forewing pattern (Fig. 2O). The close relationship of Endotheniini to Bactrini was recognized by Dang [26], based on *Endothenia*. He suggested two synapomorphies in the male genitalia: a convex, decurved tegumen, and an unusual condition in which the dorsal edge of the basal opening (or basal cavity) and the basal portion of the costa of the valva are confluent.. Dang also argued that the set of secondary sexual characters used by Falkovitsh [84] to define the tribe Olethreutini, as well as the typical distal setae of the tarsi in both sexes of that tribe, had been secondarily lost in *Endothenia* and *Bactra*. He therefore transferred *Endothenia* to Bactrini. Although Horak & R. Brown [24] and J. Brown [1] maintained the previous definition of Endotheniini, Horak [27], [29] accepted the synonymy with Bactrini. Bactrini s.s. are adapted to grasslands associated with mesic habitats, where their hosts are dominant; the larvae are stem borers in Poaceae, Juncaceae and Cyperaceae (94% of records; [56]). The 52 species in Endotheniini are typically borers in stems, roots, flowers or seeds of herbaceous plants, especially in the clade Asteridae. Bactrini s.s. are a cosmopolitan but mainly Old World group of 113 species.

While in some respects our results agree with previous ideas on the “Olethreutidii clade,” the placement of Bactrini+Endotheniini is a point of conflict. Prior phylogenies (Fig. 1) that include Bactrini as a terminal taxon portray that tribe (and sometimes Endotheniini) as branching off early from the remainder of “Olethreutidii”, leaving Olethreutini s.s. in a relatively derived position. Horak [29] stated that “Obvious similarities in the genitalia and shared reduction of tarsal setae suggest a close relationship between Gatesclarkeanini [formerly considered to be at the base of Olethreutinae but included by Horak [29] within Olethreutini following the *Neopotamia* group], *Endothenia*, and Bactrini.” Our results strongly place *Bactra*+*Endothenia* as deeply nested within Olethreutini as currently defined (**nodes 46, 45**; BP = 100, 84), rendering the latter paraphyletic. This finding is consistent with the lack of clear synapomorphies for Olethreutini. Olethreutini as recently delimited are a cosmopolitan group of 1,077 species whose larvae are typically leaf rollers.

The strong evidence for paraphyly of Olethreutini requires either that the tribe be subdivided into multiple monophyletic tribes, or that its definition be broadened to eliminate the source of paraphyly. For several reasons, we take the latter course, and hereby formally synonymize Bactrini and Endotheniini with Olethreutini. Horak [27] foreshadowed this conclusion. The present limited taxon sample was designed to estimate relationships among tribes, and provides little new information on structure within them. Current understanding of the taxa belonging to “Olethreutidii” is so incomplete that an attempt to break it into monophyletic tribes would leave many genera incertae sedis. Conversely, a broadened definition of Olethreutini would immediately place most genera (though not all; see below) confidently in a monophyletic tribe. With further research, an increasing number of clearly-defined subtribes [97] or generic groupings (sensu Horak [27]) should emerge, along with better understanding of their relationships. At that point it might be desirable to (re-) elevate the rank of those sub-clades, and to break up, or elevate in rank, the Olethreutini sensu novo proposed here. The Diakonoff subtribe Neopotamiae exemplifies recent progress in this direction. Recent study of African and related taxa has clarified the content of this group and yielded a striking synapomorphy supporting its monophyly, a characteristic signum in the female genitalia consisting of a single plate with 1–3 anteriorly directed projections [98], [99]. Furthermore, the present study, in which Neopotamiae are represented by *Afroploce* (Fig. 2N), provides strong evidence that this subtribe is related to Bactrini+Endotheniini (**node 47**; BP = 100), and is possibly even the sister group of these (**node 48**; BP = 62). Several shared morphological characters of Bactrini and the Gatesclarkeana and Neopotamia groups are discussed by Horak [27]. These related clades differ markedly in the prevalence of male secondary sexual characteristics such as ancillary scent organs, which are widespread in Neopotamiae but nearly absent in Bactrini+Endotheniini.

The exact limits of Olethreutini sensu novo remain tentative, even in our own results. The grouping of *Episimus* and *Oxysemaphora* with the other Olethreutini sampled is very weakly supported (**nodes 43, 44**; BP<50), and these taxa fall elsewhere on the tree, also with little support, when synonymous change is excluded. *Episimus* is an enigmatic genus which Diakonoff [97] did not try to place in his classification, but *Oxysemaphora* was placed in his “Olethreutae.” Horak [27] tentatively includes both genera in the *Oxysemaphora* group at the base of Olethreutini, acknowledging the numerous plesiomorphic characters. Thus, further instances of paraphyly of Olethreutini, with respect to Enarmoniini+Eucosmini+Grapholitini [24], [29] or other taxa, cannot be confidently ruled out, and further adjustments may be necessary.

### Molecular Phylogeny: Summary and Conclusions

The molecular analyses presented here yield a new working hypothesis of relationships among the tribes and subfamily of Torticidae (Fig. 3) in which the great majority of inferred groupings (82%) are strongly supported (≥80% bootstrap). The molecular phylogeny mostly upholds previous hypotheses, but not always. Its major features are as follows:

1. The subfamilies Tortricinae and Olethreutinae are each monophyletic, and are sister groups. Previously, Tortricinae had been hypothesized to be para- or polyphyletic.
2. Chlidanotinae are confirmed to comprise the earliest-diverging tortricid lineages. In contrast to morphology, the present molecular data moderately favor paraphyly for this subfamily, with Polyorthini as sister group to all other tortricids. This result varies with outgroup and character choice, however, and no strong conclusion can be drawn.
3. Within Tortricinae, Phricanthini diverge first, while the remaining tribes form two strongly supported sister groups: (Sparganothini+Atteriini)+(Ceracini+Archipini), and (Cnephasiini+Tortricini)+(Euliini+Cochylini). Euliini are paraphyletic with respect to, and are here synonymized with, Cochylini.
4. Within Olethreutinae, Microcorsini diverge first, while the remaining tribes fall into two lineages. One of these contains the strongly supported group (Eucosmini+Grapholitini), joined with very weak support by Enarmoniini. The other consists of Olethreutini+(Bactrini+Endotheniini), with the latter group nested inside, and here synonymized with, the former. The limits of Olethreutini as thus redefined are tentative, as the association of two early-diverging genera with the remainder is very weakly supported (<50% bootstrap).

### Evolutionary trends in life history and distribution

The new phylogenetic framework permits re-examination of several conjectures about life history evolution and biogeography in Tortricidae. To facilitate such a review, we present in Figure 5 a provisional synopsis of species diversity, prevailing larval feeding habits and geographic distribution for the tribes included in this study, synthesized from Brown et al. [56], Janzen and Hallwachs [57], and other sources. These are superimposed on a simplified version of the molecular phylogeny, in the manner of Powell et al. [33], with tribes as the terminal taxa. This overview has multiple limitations, among which are the paucity of observations for some tribes, the absence of three tribes from our sampling, and ambiguity in the interpretation of some host records (see Methods). For these reasons we do not attempt any formal comparative analyses, and aim mainly to help to identify the most promising directions, and most critical observations to be made, for future more detailed studies.

There have been multiple conjectures on the ancestral larval feeding habits of Tortricidae [33]. The idea that the most basal tortricids belong to Tortricinae, which include several saprophagous groups, led to the suggestion the family initially fed on detritus or fungi [24]. In the new phylogeny, however, the basal lineages of the family as a whole (i.e., Chlidanotinae), as well as of Tortricinae (i.e., Phricanthini) and Olethreutinae (i.e., Microcorsini), are all phytophagous so far as is known, very strongly suggesting that this was the ancestral tortricid condition. Subsequent shifts to saprophagy or mycophagy have apparently occurred multiple times, e.g., in Tortricinae: Epitymbiini (not included here) and some Euliini.

For phytophagous tortricids, it has previously been postulated that the ancestral habit was external feeding, albeit from within a rolled leaf or other shelter, as is typical for external feeders outside the Macroheterocera [24], [33]. The new phylogeny, in contrast, suggests that the ancestor is equally likely to have been an internal feeder; the data available do not support a confident choice between these alternatives. Observations on Chlidanotini and Hilarographini are few, but suggest that both tribes are borers as larvae, in twigs, fruits or seeds. On the other hand, both internal feeding and leaf rolling have been reported from the most basal lineage, Polyorthini, and there is no information as to which is more primitive. However, an anal comb is present in all known polyorthine larvae, suggesting external feeding as this structure serves to eject feces from the larval feeding shelter. Appeal to outgroups does not help, as no other lepidopteran lineage is obviously closely related to Tortricidae.

Limited life history evidence, long branches and repeated transitions between internal and external feeding may preclude us from ever confidently deciding whether or not Tortricidae were ancestrally internal feeders. However, there is clear evidence within the family for broad-scale evolutionary trends in this character, as well as extensive homoplasy (Fig. 5). External feeding was probably ancestral for Tortricinae (**node 8**), in which nearly all the tribes, including the earliest-branching (Phricanthini), consist entirely or mainly of external feeders. The only major internal-feeding tortricine lineage is Cochylini sensu stricto, which probably evolved from an externally-feeding ancestor, given its highly derived cladistic position (**nodes 21–24**). In contrast, Olethreutinae (**node 7**) show much more even representation of, and more frequent apparent transition between, internal and external feeding. Although the oldest lineage, Microcorsini, feeds internally, the ancestral state for the subfamily is not obvious, as the earliest-branching lineages within both major subdivisions of the remaining olethreutines (**nodes 31, 43**) are partially or entirely external feeding. Repeated shift in habit is evidenced by the fact that two independent tribes (Enarmoniini, Eucosmini) show substantial proportions of both internal and external feeders. Moreover, both major subdivisions of olethreutines (**nodes 31, 43**) include a predominantly internally-feeding lineage (Grapholitini; Bactrini+Endotheniini) which is sister-group to and/or nested within an externally-feeding lineage. Two recent studies suggest that external-feeding phytophagous insect lineages may consistently undergo greater diversification than their internal-feeding relatives [100], [101]. Given the patterns documented here, it appears that Tortricidae can potentially contribute important evidence on this hypothesis. For Olethreutinae, internal feeding appears to have favored diversification, at least in the *Eucosma*-*Epiblema* group of genera, all with known larva being internal feeders, as well as in *Dichrorampha* and many other Grapholitini.

Previous authors have also addressed the evolution of host range and its correlation with other life history traits. As is evident from our compilation (Fig. 5), host range varies widely within many tribes and genera of Tortricidae, but also shows broader phylogenetic trends. In Chlidanotinae and Olethreutinae, oligophagy as defined and measured here (see Methods) is the predominant habit in nearly all tribes. In Tortricinae, on the other hand, polyphagy predominates in seven of the nine tribes studied. Within Tortricinae, relative prevalence of polyphagy is consistent with Powell's hypothesis [20] of an association with oviposition in large clusters. Deposition of eggs singly or in small scattered groups, found in most tortricid tribes, is probably the ancestral condition both in Tortricinae and in the family as a whole. Oviposition in large clusters is restricted to, and may have arisen in the ancestor of, the clade consisting of Atterini+Sparganothini and Ceracini+Archipini (**node 10**). All four tribes in that clade also have higher estimated proportions of polyphagous species (69–100%) than all other tortricine tribes, and indeed all other tribes in Tortricidae except Microcorsini. There is also an apparent correlation of host range with feeding mode. Both across Tortricidae as a whole, and within the two largest subfamilies, tribes which are entirely or mainly internal feeders almost invariably have lower fractions of polyphagous species than any of the tribes which feed outside the plant, mostly as leaf rollers or tiers. The only exceptions to this trend are the earliest-diverging (and relatively little-studied) tribes in both Olethreutinae and Tortricinae, namely the Microcorsini, whose larvae feed inside seeds or fruits, and the Phricanthini, which appear to live on bark or leaves. This ostensible pattern merits detailed examination in future studies, as a linkage between internal feeding and restricted host range has been repeatedly postulated but rarely tested empirically [100], [101].

Previous authors have also remarked, finally, on the mainly southern geographic distribution of several putatively plesiomorphic tortricid tribes or genera, suggesting that these taxa represent relicts from an ancestral Gondwanan radiation of Tortricidae [29], [102], [103]. The new phylogeny (Fig. 5) appears at least consistent with this idea. The earliest-branching tribes in the family as a whole, as well as within the two largest subfamilies –Polyorthini, Chlidanotini, Hilarographini, Phricanthini and Microcorsini – are all relatively species-poor groups with mainly southern/tropical distributions. The cosmopolitan distributions typical of the other tribes, especially the larger ones, with substantial representation in temperate regions, appear to be derived. Additional evidence, including fossil-calibrated divergence dating, is needed to further test the Gondwanan-origin hypothesis.

## Supporting Information

Table S1 **List of specimens sampled, including collection localities, LepTree voucher identification numbers and codes, and GenBank accession numbers.**
(XLS)
